# Multiple seasonality in soil radon time series

**DOI:** 10.1038/s41598-019-44875-z

**Published:** 2019-06-13

**Authors:** Marianna Siino, Salvatore Scudero, Valentina Cannelli, Antonio Piersanti, Antonino D’Alessandro

**Affiliations:** Istituto Nazionale di Geofisica e Vulcanologia, Rome, Italy

**Keywords:** Geophysics, Geochemistry

## Abstract

The dynamics governing the movement of the radon are complex and dependent on many factors. In the present study, we characterise the nature of temporal variations of 2-hourly and daily radon measurements in several monitoring sites of the Italian Radon mOnitoring Network (IRON) in Italy. By means of continuous wavelet transformation, a spectral analysis in time-frequency domain is performed. The results reveal that there are sub-daily, daily and yearly persistent periodicities that are common for all the stations. We observe structural seasonal breaks, that occur at the same frequency but at distinct time. Variations in radon concentration and local temperature are studied in terms of frequency contents and synchronicity. When analysing several long time series together, it is evident that the phase difference at low frequency movements (365-day period) between the radon and local temperature time series is depending on the sites’ location and therefore strongly controlled by local factors. This could at least partially explain the apparently contrasting results available in the literature obtained investigating smaller dataset about the relationships between temperature and radon variations. On the other hand, results show that all radon time series are characterised by marked cycles at 1 and 365-days and less evident cycles at 0.5-day and 180-days. They would be all ascribable to environmental-climatic factors: the short-period cycles to temperature and pressure variations, the long-period cycles also to seasonal rainfall variations.

## Introduction

Radon (^222^*Rn*) is a radioactive gas with a half-life of 3.8 days. Radon originates in the natural decay of other radioactive elements in the Earth’s crust; it is a noble gas and extremely stable, without relevant interaction with other elements. The transport of radon in soil or water is mainly performed by other gases or fluids (e.g. CO_2_) acting as carriers.

The monitoring of soil radon emission is today a topic of interest because of the risk that this element poses to human health but also for its relationship with several geological-environmental processes. The World Health Organization (WHO, http://www.who.int/en/) and the International Agency for Research on Cancer (IARC, http://www.iarc.fr/) have classified radon as a class 1 carcinogenic factor and it is considered by the scientific community the second cause of lung cancer after cigarette smoke. The relatioships between radon and the environmental processes concern the volcanic activity, tracing in atmospheric, marine and hydro-geological issues, it is studied as a tracer of tectonic activity and earthquake precursor^1–3^. Indeed, the latter topic encounters a great interest worldwide since clues of tectonic-induced anomalies in radon signals have been suggested in several cases^2,4–8^. Such anomalies likely arise from the preparatory crust fracturing processes leading to an earthquake: the release of stress within the crust eases the migration of radon from deeper sources. Earthquake-related anomalies cannot be easily and univocally discriminated from other anomalies of different origin, and therefore the use of radon as a precursor of seismic activity is debated and should be taken with great caution.

The signals from radon sources are directly and indirectly influenced by several factors. Besides the transient geological-tectonic processes above mentioned, they are: flux of carrier gases, environmental and climatic variables, characteristics of the ground soil, tide, solar effect, etc^9–16^. Therefore, radon time series, either in the short- and in the long-term, present a complex dynamic structure and the study of the occurrence of long-range correlations and of fractal scaling properties are of great interest^17^. Some controlling factors, such as climate or tidal forces, reflect in a multiple-seasonality of the radon time series: hourly, diurnal, multi-day, and annual cycles have been detected in different worldwide studies and put in relationship with the above mentioned controlling factors^18–23^. In all the papers dealing with radon time series the behaviours of the controlling factors is not univocal and therefore their influence has been suggested to be site-specific^24^. Moreover, the effect of controlling factors acting at larger scale can be subordinated in term of amplitude and masked by primary factors (e.g. climate and geological conditions). It is noteworthy that, as a consequence of the multiple-seasonality, radon time series are also characterised by non-stationary behaviour and therefore the use of statistical methodology for stationary time series can lead to misleading conclusions^25^. Usually, the heteroscedasticity is a common features of radon time series, the variability of the measurements is not constant over time. As we consider concentrations, the Gaussian assumption is restrictive, the probability density function of the observed values is defined for positive values and often it is non-symmetric, resulting in a non-normal distribution^17^. The external factors controlling the radon time series cannot be simple expressed by linear stochastic processes, but require the specification of non-linear processes with explicit terms for the dependence to external factors.

In this paper, we use descriptive statistics to characterise the radon time series in particular the seasonal behaviour and the variability along the year. Furthermore, we investigate the multi-periodicity behaviour in 2-hours and average daily radon time series recorded at nine selected sites belonging to Italian Radon mOnitoring Network (IRON^26^). The soil radon emission is a topic of great concern in Italy where the mean annual concentration has been estimated to 70 Bq/m^3^, higher with respect the global annual mean of 40 Bq/m^3^ ^27^ and also because the recent seismic sequences occurred in Italy represented a good opportunity to investigate the relationship between soil radon emission and earthquakes^24,28^. We perform a power spectral analysis in time-frequency domains using continuous wavelet transformation (CWT)^29^. The different time series are characterised according their underlying long-memory correlation structure. Moreover, we investigate the potential influence of the local temperature, measured by a sensor co-located with the radon detector, using different types of wavelet transformations (i.e. the cross-wavelet power density and the phase difference).

## Data

We consider nine radon monitoring sites belonging to IRON, nowadays a network with near 50 permanent stations covering a wide area of Italy which installation started in 2009. The network is mainly designed for monitoring the radon concentration in tectonically active areas, in order to investigate the possible relationship between local seismicity and variability in radon emissions^26,28,30^.

Since we want to describe a long-range behaviour of radon emissions, we select a subset of monitoring stations that have the greatest number of continuous measurements.

The selected stations are equipped with a Lucas cell, an alpha scintillation detector with an acquisition window of about 2 hours (115 minutes of data acquisition followed by a 5 minutes standby time). The Lucas cell consists in a flask, which inner surface is coated with silver-activated zinc sulphide (ZnS). It integrates a front-end electronics and measures radon concentration by counting the radon decay signals in the given acquisition window. Besides, all the stations are equipped with a local temperature sensor characterised by an acquisition window of 1 hour. Mainly, for each monitoring site we focus on four time series: 2-hourly and average daily radon measurements, and 2-hourly average and mean daily local temperature.

**Figure 1:**
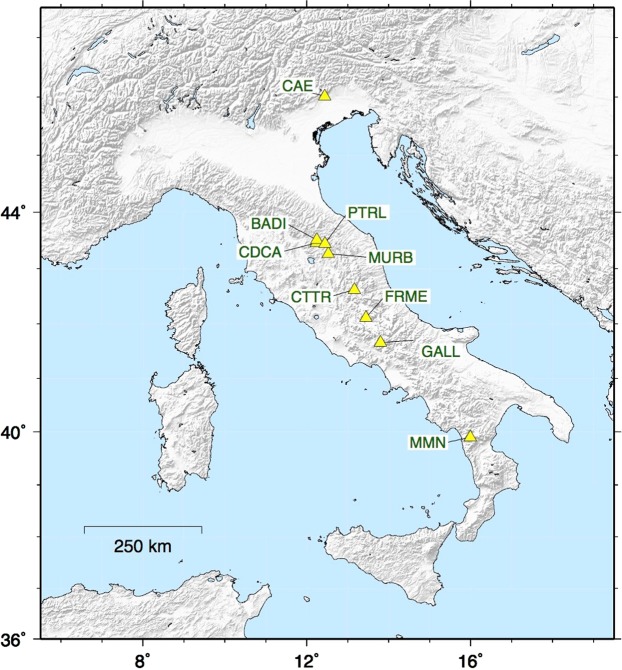
Italy map and locations of the selected radon monitoring stations of the IRON network.

The map of Italy in Fig. 1 shows the location of the selected stations. The raw data of 2-hourly radon time series of the nine selected stations are in Figure S1. The Table 1 summarises the details of radon monitoring sites in terms of geographical coordinates, altitude, time span, and length in days. Moreover, the installation type of each station is indicated since it could influence the fluctuation of radon measurements and its relationship with other external factors^26^. In the “Indoor” stations, the instrument is located in the basement of a building, typically in a closed technical room not usually accessed by people. In the “Shelter” stations, the instrument is located in a small shelter usually together with a seismic and/or geodetic station. In the “Borehole” stations, the instrument is installed in a shallow borehole usually no more than 2 meters deep. When possible, the installations in shelter or borehole are preferred because they should reduce the impact of temperature, atmospheric pressure, and rainfall on the measurements, allowing a better identification of the potential radon anomalies^26^.

**Table 1:** Main information of the selected monitoring sites. The type of installation can be “B = Borehole”, “I = Indoor” and “S = Shelter”^26^. *t*_1_ and *t*_*n*_ indicte the observation period considered in the analysis. *n*_*day*_ is the length of the time series in days.

Code	Location	Type	Lat	Long	Alt	*t*_1_	*t*_*n*_	*n*_*day*_
BADI	Badiali-PG	S	43.51	12.24	430	28/08/2014	23/05/2017	1000
CAE	Caneva-PN	B	46.01	12.44	870	04/08/2015	31/08/2016	393
CDCA	Citta’ di Castello-PG	S	43.46	12.23	50	23/01/2014	20/11/2017	1398
CTTR	Cittareale-RI	I	42.62	13.16	960	14/05/2010	17/07/2015	1891
FRME	Forme-AQ	I	42.11	13.44	1012	18/06/2013	23/12/2015	919
GALL	Gallinaro-FR	I	41.67	13.82	452	20/03/2015	31/01/2017	684
MMN	Mormanno Faro-CS	I	39.90	15.99	921	08/12/2011	03/10/2016	1762
MURB	Monte Urbino-PG	B	43.26	12.52	854	09/07/2013	26/07/2016	1114
PTRL	Pietralunga-PG	I	43.44	12.44	750	28/09/2012	01/08/2015	1038

## Results

### Descriptive analysis

The Table 2 summarises the daily radon time-series (see Figure S2) from the nine selected monitoring sites with some descriptive statistics. As reported by the coefficient of variation, the daily time series recorded in BADI, CTTR, and GALL show less variability. According to the skewness and the kurtosis coefficients, all the daily radon time series do not follow a Gaussian distribution.

**Table 2:** Descriptive statistics of daily radon concentration time series for each selected site (values are in Bq/m^3^). In particular, CV is the coefficient of variation, a measure of relative variability. %*NA* is the percentage of missing values. *ρ*(*Rn*, *Temp*) is the estimated Pearson’s correlation coefficient between average daily radon measurements and local temperature. (*) highlights if the correlation coefficient is statistically different from zero at 5% significance level.

	BADI	CAE	CDCA	CTTR	FRME	GALL	MMN	MURB	PTRL
Mean	39.33	208.35	47.02	169.04	58.71	62.96	241.13	25.67	138.59
Max	80.39	1116.07	433.99	377.61	543.77	98.31	1581.04	388.05	377.86
Min	23.92	32.60	3.98	74.59	15.29	21.90	6.18	1.78	20.35
CV	0.18	0.77	0.66	0.27	0.60	0.22	1.08	1.47	0.43
Skewness	0.94	0.94	4.17	1.02	3.76	−0.25	1.75	4.44	0.44
Kurtosis	2.35	2.38	33.36	1.46	40.83	−0.24	2.75	25.19	−0.03
%NA	2.70	0	1.64	0.37	2.83	3.95	1.30	0.90	0.87
*ρ*(*Rn*, *Temp*)	0.37(*)	−0.64(*)	0.00	0.39(*)	−0.06	0.39(*)	−0.17(*)	0.33(*)	0.55(*)

The relationship between the radon measurements and the local temperature is measured with the Pearson’s correlation coefficient (*ρ*) and the results are site specific, not generalisable. In BADI, CTTR, GALL, MURB and PTRL, there is a significant positive correlation, in CAE and MMN is negative, and in CDCA and FRME is not significant. This behaviour is also confirmed by the 15-days moving average radon and local temperature time series in Figure S3. All the 15-days moving average radon time series show a rough seasonal pattern and, as an obvious consequence, it seems to be connected with the local temperature variations (with a positive or negative correlation depending on the site).

**Figure 2:**
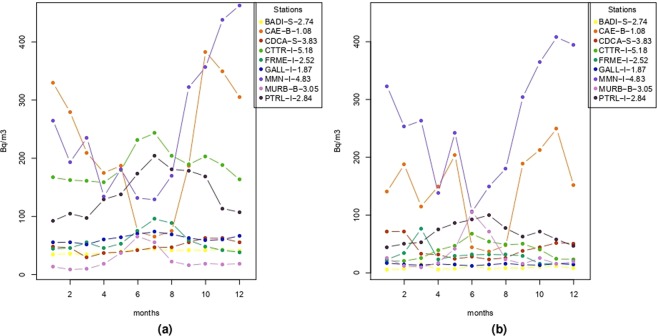
(**a**) For each station, the colored line joins the radon monthly average concentrations. (**b**) The lines indicate the radon monthly standard deviation. In the legend, there are the station code, the type of installation (“B” is borehold, “I” is indoor and “S” indicates shelter installation) and the time series length in years.

To investigate the seasonal behaviour, for each site, the monthly means are computed, see Fig. 2(a). The stations located in shelter (BADI and CDCA) show a less evident seasonal variations if compared with the other stations. Indoor stations are characterised by a marked seasonality over the annual cycle, except for GALL, which trend is comparable to the shelter type. In particular, CAE and MMN are the sites characterised by the higher variation in the monthly means; also the MURB borehole station shows a seasonal variation. However, the trends shown in Fig. 2 should be considered indicative since they result from time series with different length. The standard deviation of the monthly time series for each station are in Fig. 2(b).

**Figure 3:**
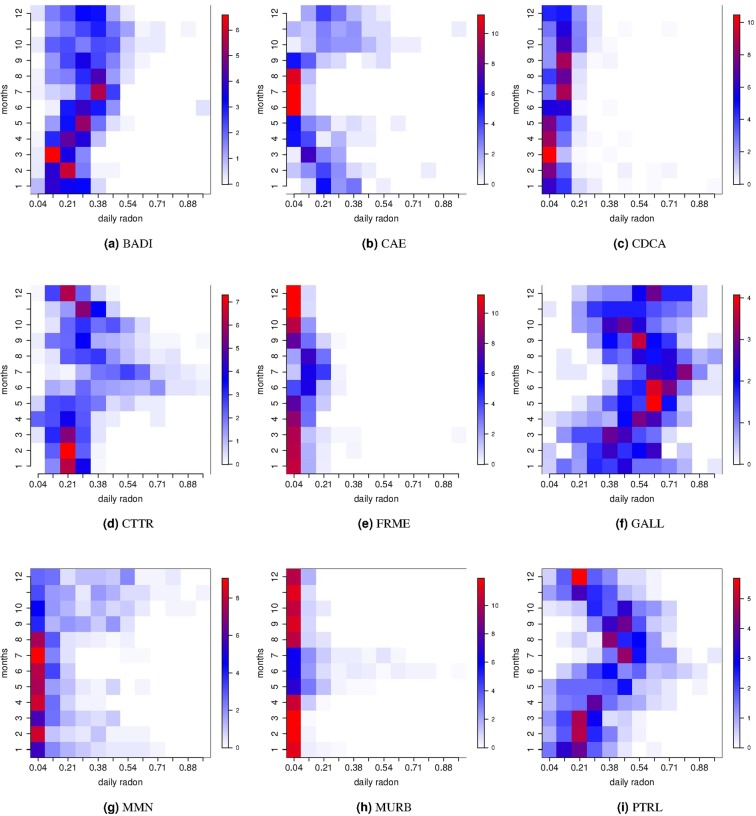
For each site, the conditional monthly probability density function (conditional histogram) of the standardised daily radon is plotted. In the vertical axis, there is the month index and in the horizontal axis there is the standardised daily radon concentration ranging between 0 and 1. For a fixed month, red (white) colour indicates radon intervals with higher (lower) density.

Additional information in terms of variability are instead provided by Fig. 3, showing the conditional monthly histogram of the standardised daily radon measurements in [0, 1] (for each site, the radon observations have been standardised subtracting the minimum, and then dividing by the range). In CAE, CDCA, and MMN during the summer period the radon measurements assume lower values and are less variable than the other months. In the other stations, the seasonal pattern show an opposite general trend with higher values and variability during the summer period. Remarkably these temperature positively correlated stations, following Fig. 3 patterns are characterised by a more complex behaviour with respect to anticorrelated ones (except MMN). We remind that the positive correlation with temperature has been more frequently observed in other investigations^9,30–32^. CDCA, FRME, and MURB are the sites where measurements are more stable in terms of both mean and variability over the months, conversely, the other stations are characterised by a not costant variability of radon concentration over the months.

### Power spectrum densities

We performed the spectral analysis for both 2-hourly and average daily radon measurements. The results for the 2-hourly data are in the supplementary section. and the Figure S4 shows the image plots of the wavelet power spectrum (WPS, Equation (3)). The period on the vertical axis ranges from 4-hours to 16-days, the time-frequency regions with warm colours represents areas with high power, the black lines indicate the significant maxima of the undulations of the wavelet power spectrum, and they give an indication of the permanent cycle period.

Clearly the series exhibit transient dynamics and the magnitude of the WPS is not constant over time fixing a specific frequency. In particular, we can observe that for all the series there is a high value of the spectral power density at about 1-day periodicity but this periodicity is not persistently significant (with a 10% significant level). This evidence is not trivial since the diurnal cycle is universally considered the most clear periodicity in radon concentrations time series. As far as the whole high-frequency spectrum is considered, for CTTR and PTRL series the WPS assumes lower values for all the observed autumn-winter periods. Similar behaviour is for the FRME series just for the first years. Instead, the BADI series has a lower WPS just in the winter period. On the other hand, CDCA and MMN series behave in the opposite way, they have lower WPS value during the spring-summer period and CAE series just for the summer period thus confirming a transient dynamics and a complex behaviour. Additionally, for each image plot the global wavelet power spectrum averaging over time is plotted in Figure S5. The horizontal lines provide references at half-day, one-day, and seven-days periods. Each site is characterised by one, or more peaks. As expected, all the time series have a clear 1-day periodicity and it is the most prominent feature at several stations. There is no evidence of notable, longer periodicities shared between the various sites. Conversely is recognisable a weak periodicity at 0.5 day sometimes masked within the 1-day greater peak.

**Figure 4:**
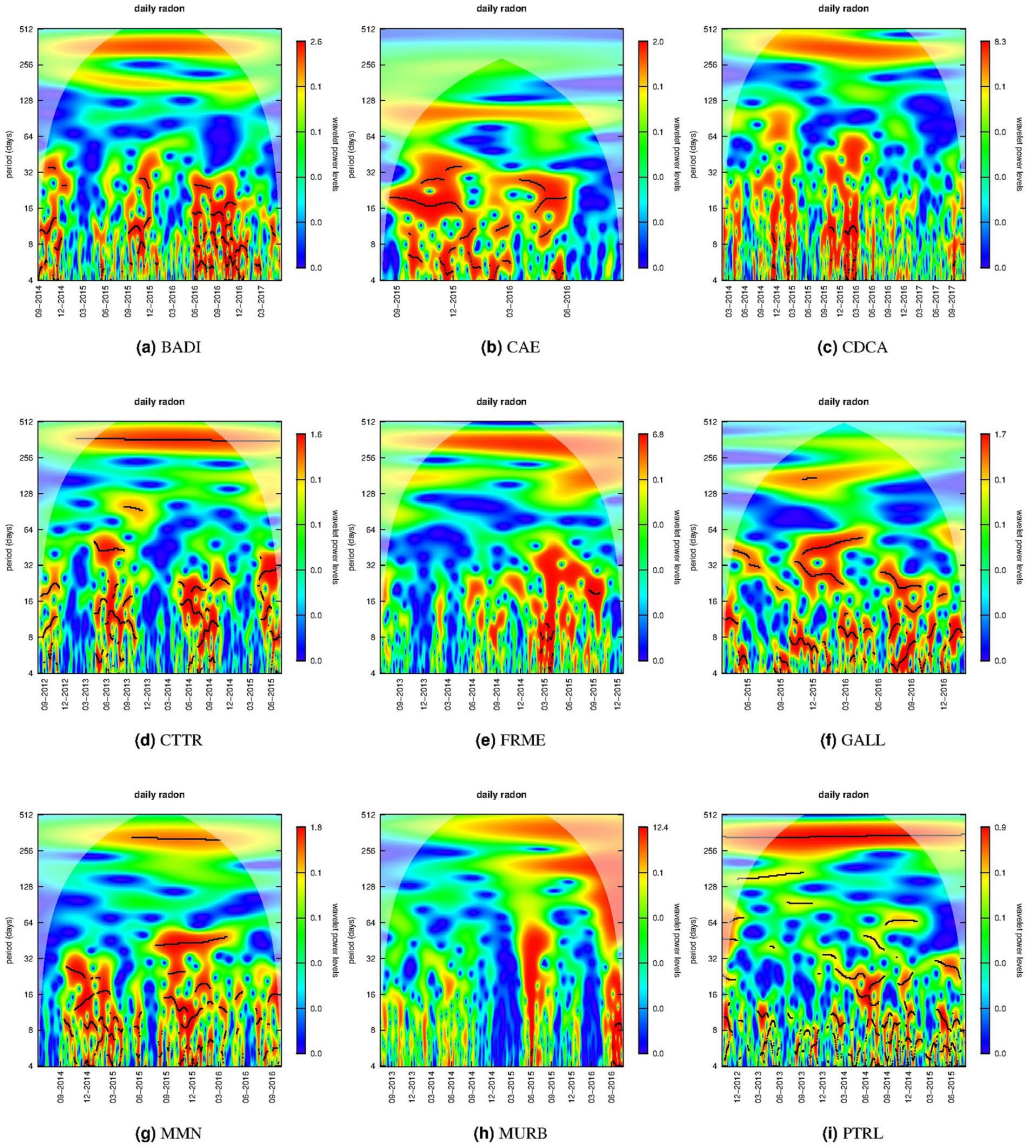
Wavelet power spectrum of daily radon series in time-frequency domain with the CWT method. The black contour indicates the significant period with 90% confidence level. The lighter shade is the regions influenced by edge effects. The corresponding power spectrum density marginalising over time is in Fig. 5.

**Figure 5:**
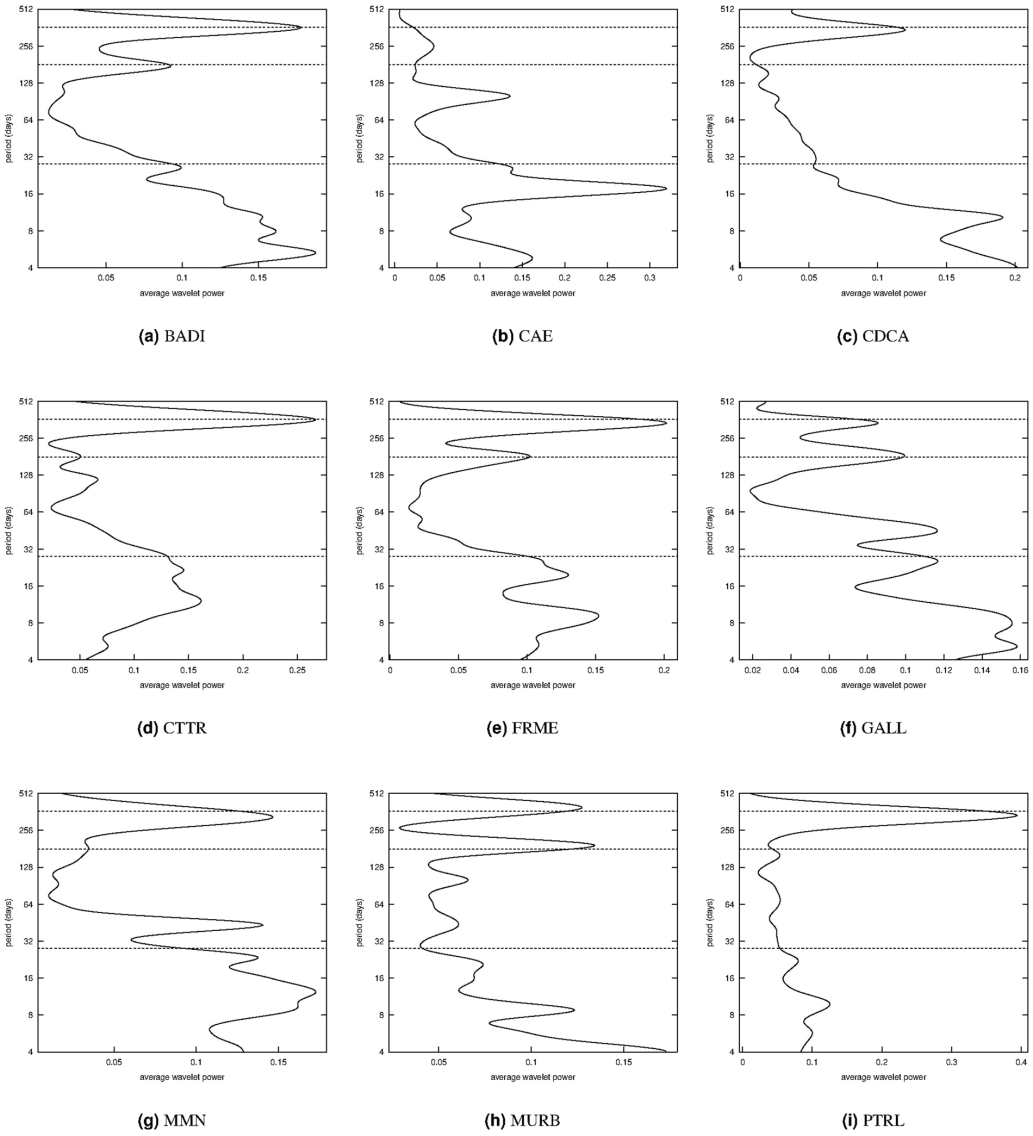
Global power spectrum density for mean daily radon time series. The horizontal lines are for the 28-day, 180-day and 365-day periods.

For the daily radon data, the WPS is in Fig. 4 where the period ranges from 4 to 512 days. The thick black contour indicates the 90% confidence level and the lighter shade indicates regions inside the cone of influence due to the border effect. The Fig. 5 shows the global wavelet power spectrum; the horizontal lines provide reference at 28, 180, and 365 days. All the series (except CAE) show a clear 1-year periodicity that, differently from 1-day periodicity, is persistent along time. A subordinate periodicity at about 180 days results in all the stations apart CDCA.

It may be remarked that the CAE time series spans only about ~400 days because the temperature data were not available (Figures S1 and S3). For this reason the 180-days and 365-days cycles are undetectable, they appear when considering the whole available radon dataset in Figure S1 (and this result is not shown), but disappear when the cut is performed (Fig. 5). For shorter periods some stations have marked peaks (eg. CAE at about 16 days; CDCA and MURB at about 10 days) but they are not common among the various sites.

### Cross-wavelet power and phase difference

In order to examine the relationships between radon time series and local temperature, the global cross-wavelet power (time-average of Equation 5) and the phase difference (Equation 6) are computed. In Figure S6, the global cross-wavelet power spectrum, that can be interpreted as the covariance between the two time series, is computed from 4-hours to 16-days based on the two-hourly time series. At almost all the stations, the two series have joint periods at 0.5-day and 1-day, since the global cross-wavelet power has peaks at these frequencies.

**Figure 6:**
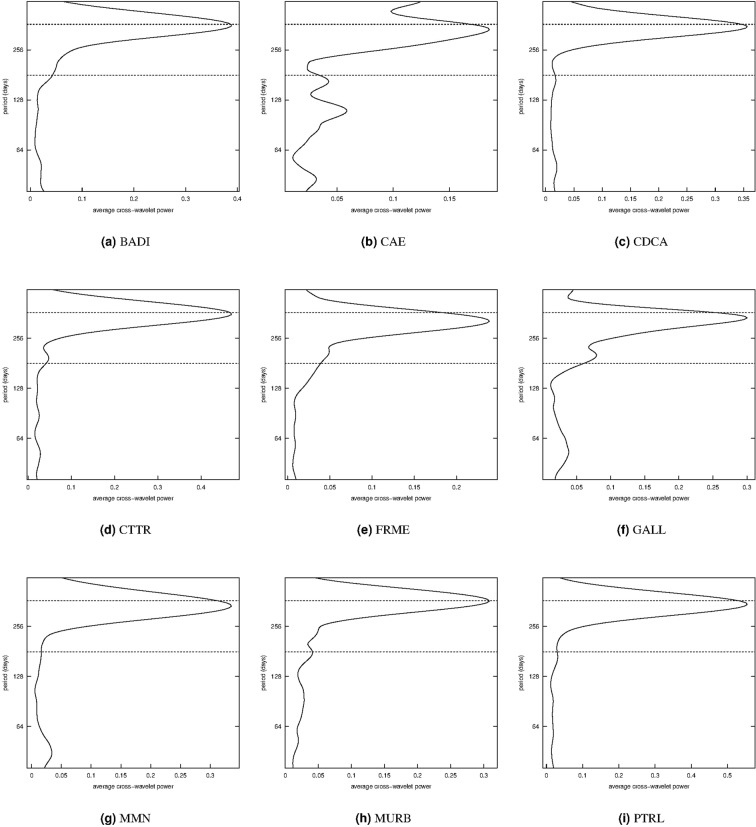
Global cross-wavelet power spectrum of mean daily radon and temperature time series. The horizontal lines are for the 180-day and 365-day periods.

**Figure 7:**
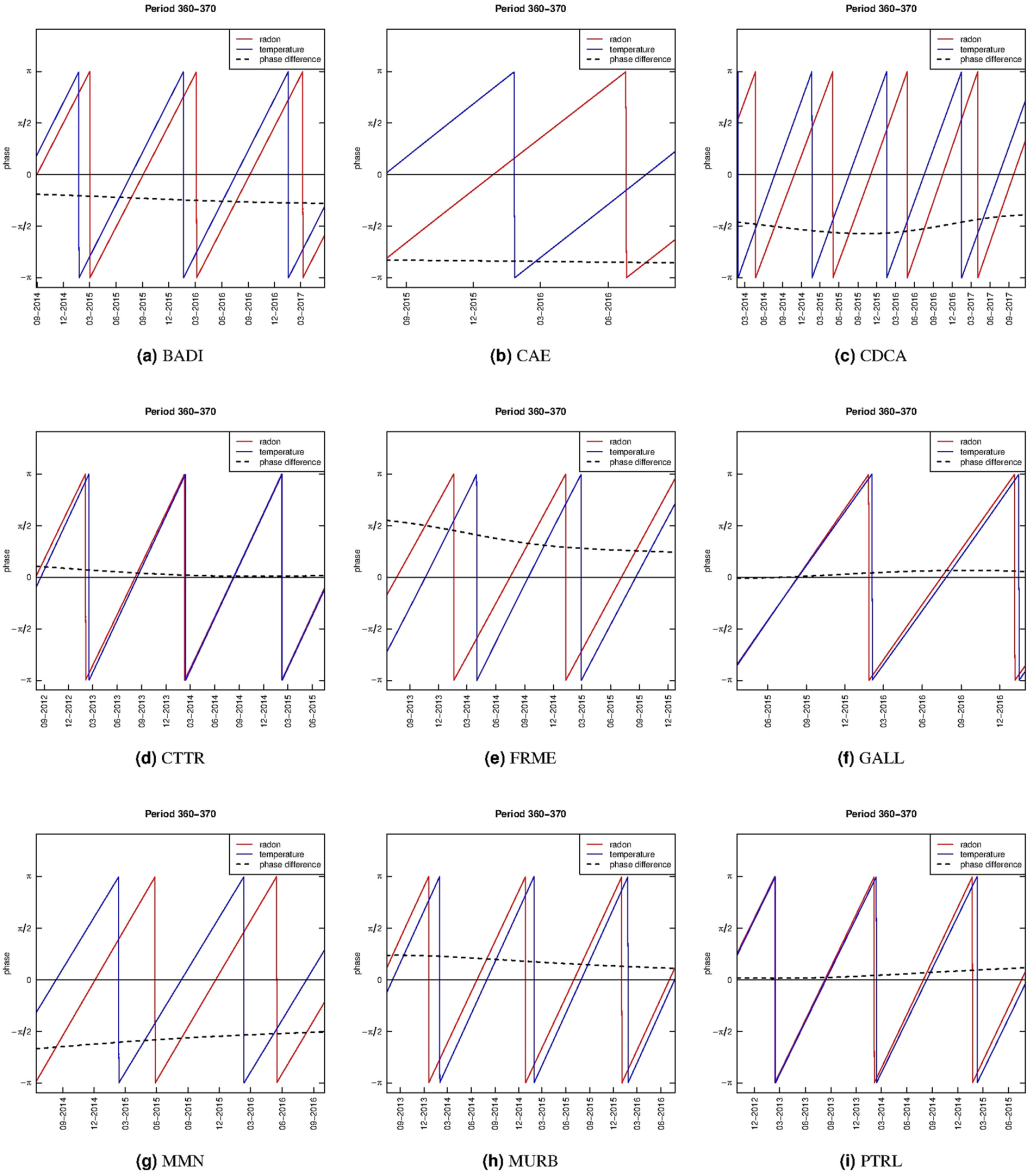
Phases and phase differences between radon and local temperature time series at the range period 360~370 days.

On the other hand, based on the daily measurements, the corresponding global cross-wavelet power spectrum is in Fig. 6 with period ranging from 16 to 512 days. The local temperature is highly correlated with radon around the 365-days cycle. Therefore, for the 360~370 day band, the phase of the daily radon measurements and the local temperature are computed. In Fig. 7, the red lines are the radon phase at every station, the blue lines represent the temperature phase, and the dashed black lines are the phase differences. We can see that for the CTTR, GALL and PTRL series, the phase-difference is zero indicating that the two series move together with no noticeable lead or delay at 360~370 day period band along time. The two series are out of phase (negatively correlated) at the CAE and MMN monitoring sites. For the BADI, MURB, and CDCA series, the two series are in phase (positively correlated) because the phase difference is between −*π*/2 and *π*/2 and this behaviour is constant over time. Instead, for the FRME series, there is a change of the phase difference in time. At all the stations (with the exception of FRME), the phase difference between radon and local temperture measurements at around 365-day period is constant, or almost constant, over time. This result suggests that the series, synchronisation between radon and temperature is stationary over time.

## Discussion

The analysis in the time-frequency domain is very common in radon time series, and also the continuous wavelet transformation has been recently applied to highlight periodicity with multi-time-resolution^2,23^. Even though cycles are clearly detected, the causative physical mechanisms governing these processes have not been fully understood. In this work, the results obtained from the analysis of the radon concentration time series present interesting features.

The descriptive analysis (Table 2) indicates that some sites (CAE, CTTR, MMN and PTRL) have a daily average greater than the Italian mean and some sites present a higher variability in radon measurements (i.e. CAE, MMN and MURB). Conditioning to the months, the monthly mean and conditional histograms are computed in order to characterise the seasonal behaviour, and they are in Figs 2 and 3, respectively. Accordingly to these results, the monthly averages and variabilities of radon concentration are not constant along the months and the observed patterns are site specific. Besides, descriptive analyses highlight also complex and site dependent patterns in the relationships between radon and temperature.

The power spectral density calculated from the 2-hourly series shows a main peak indicating a marked period at 1 day for all the nine selected sites. In the literature, this periodicity is consolidated and recognisable in the most of the radon time series, and it is due to the effect of the diurnal pressure and temperature cycle^9,11,33,34^. Intuitively radon sensor deployed in shelter and specially in boreholes should be less sensitive to climatic effects, but such rule is not unequivocal in the analysed time series (Figure S5). In the literature also the semi-diurnal cycle is encountered in most of the radon series^8,11,14,30,33,34^ and mainly correlated to the semi-diurnal atmospheric pressure effects, often referred as barometric tide^35^. The occurrence of a significant effect due to pressure is not surprising. In fact, the air circulation is a key element in the transport process of radon especially for the indoor sensors, but also the circulation in porous soils is influenced. Changes in atmospheric pressure and temperature reflect on the airflow and therefore in the radon variations; the magnitude of their effects is strictly site-specific^10^. In general, the installation in shelter or at depth should reduce such effects. We observed that the installation type did not affect the amplitude of the 1-day cycle, therefore we can likely exclude that it could affect also the semi-diurnal cycle. Therefore, the low semi-diurnal signals could be only due to the weaker semi-diurnal cycle with respect the diurnal one^35^.

No other peaks in power spectrum density for the two-hourly radon measurements (Figure S5) are shared among all the sites. Four stations (CDCA, MMN, MURB, and PTRL) show a periodicity at ~10 days: we incidentally observe that three of them are in the same geographic and tectonic area (The Alto Tiberina Fault zone) and this would suggest a common cause but presently we are not able confirm this hypothesis. All the other cycles recognisable in Figure S5 are probably site related effects, the luni-solar gravitational influence resulting in a tidal effect on radon signals has been frequently observed. The main cycles are reported at ~14.5 and ~29 days^18,36^ but also shorter periods at ~0.5, ~1, and ~7 days have been reported^12,20,37,38^ and correspond to some of the harmonic tidal constituents. The amplitude of those signals could have a relevant variability depending mainly from the geographical location but also from the underlying geology and the hydrological condition of the soil^18^. The invoked mechanism includes the direct action of the tidal attraction on the water table and on the mixture of gas contained in the rocks (i.e. CO_2_, water vapor, radon, etc) and the indirect deformation induced by the earth tide that favor the relaxing of the rocks and the opening of pathways for the transport of the mixture of gas^39^. Moreover, the cyclic variation of the gravitational forces would act as a pump, pushing the geo-fluids towards the shallower layers. The temporal lag between the tide and the radon signal at surface would be controlled by the geometry of subsurface geology and hydrogeology^18^. The power spectral density calculated from the daily series shows a main cycle at about 1 year at each station (Fig. 5) and it was fully expected considering the monthly trends in Figs 2 and 3. A subordinate cycle is present at about 180 days except in CDCA, MMN, and PTRL stations (Fig. 5); other periodicities characterize each single site. None of these last cycles correspond exactly to the 29-days period ascribable to tides; however, the periods in the range between 8 and 32 days could be related to tidal effects. The annual and semi-annual cycles on radon signals have been correlated to the annual variation of the climatic variables (mainly the temperature) in relation to the solar annual and solar semiannual cycles^19,21,23,24,32^.

The relationship between radon concentration and temperature was explored with the cross-wavelet power spectrum (Fig. 6) and phase differences in the band between 360 and 370 days (Fig. 7). The results show different values of phase difference at the various stations, but the series synchronisation at the selected frequencies is almost constant over time, even for the longer series, at CTTR and MMN. The occurrence of a 1-year cycle in the radon concentration, as well in the temperature, suggests that both the observations could be some how related to a common causative factor. The solar cycles influence the climatic variables such as temperature and pressure but also the rainfall with 180-day and 360-day cycles^40^. The precipitation is found to have a direct control on soil radon emission because the saturated ground lowers the local permeability and the radon signal is reduced^9,23,24,41,42^. Remarkably, the phase shift is peculiar at each site and almost constant (Fig. 7) and also the overall in-phase/out-of-phase relationships is site-dependent. This complex behavior could likely result from the interactions of the shift between temperature and rainfall cycles and their reflections on the convective dynamics near the surface. Therefore, we can reasonably affirm that climatic-environmental variables can account for all the observed oscillations of the investigated radon concentration time series. There is no evidence of other clear trends or anomalies but, despite all the sites are located in seismically active areas, there have been very low seismic energy emission in the time window analysed.

## Conclusions

In this paper, we analysed by means of descriptive statistics and continuous wavelet transformation the sub-daily and daily radon time series, investigating the presence of long-range memory and identifying complex dynamics and marked periodic components. Radon signals are very complex time series and require the application of advanced analysis techniques to be described thoroughly. At nine different sites belonging to IRON, we observed: i) shared daily cycles, ii) local multi-hours and multi-day signals, iii) shared periodic annual and semi-annual signals. We suggested explanation for the observed patterns, in relation with the climatic variables: variations of atmospheric pressure and temperature would be responsible for short-term periods while for long term-ones also seasonal rainfall cycles are likely to play an important role. Indeed, rainfall has also an important short term impact as widely recognised in the literature but this effect is not periodical and cannot be highlighted in the present analysis. Among periodic forcing factors, probably also the tidal forces affects the measurements and we have highlighted and discussed some possible footprints of this effect in our timeseries, but since mechanism is indirect and influenced by several local factors, the corresponding cycles cannot identified unequivocally with this type of analysis.

However, in the end, the conditions resulting from local geological and environmental settings and installation types of instrument at station are what really matter in the final recorded signal.

With respect to the results in the time-frequency domain (Figs 4 and S4) the power spectral density presents some regularities over time that seem to be dependent on the seasons. This indicates that the magnitude and variability of radon measurements change over time with a specific pattern. However to further investigate this aspect, it would be important to analyse even longer time series. For future analysis, well identified cycles, both in the short-term and in the long-term, can certainly help the comprehension of variation in radon timeseries and the characterisation of site-depended radon behaviour. The approach followed in this study is not focused to immediately detect earthquake-related anomaly, however it would turn useful because the de-noising of the series removing the identified cycles, would enhance the anomalies related to geological processes^43^. The configuration of the stations belonging to the IRON networks are conceived to minimize the effects of climatic variables (temperature, pressure, wind) on the measures without increasing the economic impact and infrastructural complexity of the monitoring stations. This approach permitted the implementation of a dense network allowing us to analyse several different time series longer than one year at the same time. This represents a novelty and an advantage when trying to assess definite regularities in such complex signals like radon time series. On the other hand, the effects of climatic variables in IRON stations time series are still important not only in the indoor stations but also in the shelter and borehole ones. This means that an improvement in the design of the station and the relative trade-off with cost and complexity should be further investigated.

To be better isolated from the climatic and environmental noise, the sensors could be placed at greater depth but available quantitative evidence on this are not univocal^8^. When located at surface, the station could be also equipped with temperature, pressure, and soil moisture sensors sampling at least at the same frequency of the radon sensor.

## Methods

In the literature, there are several proposed methods for detecting long-range memory in observed radon time series, such as the ARFIMA model and the spectral analysis, for a general overview see^17^.

Usually, the Fourier analysis is used to study time series in the frequency domain. This methodology is appropriate for stationary time series, as matter of fact the time information is lost and it is not possible to distinguish transient relations and identify structural changes. In our case, the observed time series clearly present a non-stationary behaviour (Figures S1 and S3) and the spectral analysis based on the continuous wavelet transformation (CWT) is preferred. In this paragraph, we briefly describe the spectral analysis in time-frequency domain based on the continuous wavelet transformation of univariate and bivariate time series following the notation in^29,44^. By means of this methodology, we identify and quantify the presence of long-range memory analysing a single time series and we deal with the time-frequency dependencies between two time series. We consider the space ${L}^{2}({\mathbb{R}})$, the set of square integrable functions satisfying ${\int }_{-\infty }^{+\infty }|x(t{)|}^{2}dt < \infty$, and denote by the capital letter, X(t) the Fourier transformation of a given function, $X(\omega )={\int }_{-\infty }^{+\infty }x(t){e}^{(-i\omega t)}dt$. A function *ψ*(*t*) ∈ *L*^2^(*R*) that satisfies the admissibility condition ${\rm{\Psi }}(0)={\int }_{-\infty }^{+\infty }\psi (t)dt=0$ is called “mother wavelet”, and a doubly-indexed family (“wavelet daughters”) is generated by scaling and translating *ψ*(⋅):

1 $${\psi }_{\tau ,s}(t)=|s{|}^{-\mathrm{1/2}}\psi (\frac{t-\tau }{s})$$

with $s,\tau \in {\mathbb{R}}$ and *s* ≠ 0. The CWT of a given function $x(t)\in {L}^{2}({\mathbb{R}})$ with respect to the wavelet family (1) is:

2 $${W}_{x;\psi }(\tau ,s)={\int }_{-\infty }^{+\infty }x(t)|s{|}^{-\mathrm{1/2}}{\psi }^{\ast }(\frac{t-\tau }{s})dt$$

where * represents the complex conjugate operation, *s* is the scale parameter controlling the wavelet width and *τ* controls the wavelet location in the time domain. In this analysis, we use the well-known, quite flexible and complex-valued Morlet mother wavelet that takes the form $\psi (t)={\pi }^{-\mathrm{1/4}}{e}^{i\omega t}{e}^{-{t}^{2}\mathrm{/2}}$.

Usually, with an image plot with respect to (*τ*, *s*), the (local) wavelet power spectrum (WPS) is plotted and it is computed as the squared of (2),

3 $${(WPS)}_{x}(\tau ,s)=|{W}_{x;\psi }(\tau ,s){|}^{2}$$

To do a comparison with the classical spectral method, the previous quantity can be averaged over time (*τ*) obtaining the global wavelet power spectrum, ${\int }_{-\infty }^{+\infty }|{W}_{x;\psi }(\tau ,s{)|}^{2}d\tau$. If *ψ*(⋅) is a complex function then (2) can be separated into its real (${\mathcal R} \{{W}_{x;\psi }(\tau ,s)\}$) and imaginary (${\mathcal I} \{{W}_{x;\psi }(\tau ,s)\}$) parts, and so we can have information on both local amplitude and instantaneous phase across time. In particular, the phase-angle of the complex number (2) is

4 $${\varphi }_{x}(\tau ,s)=\arctan (\frac{ {\mathcal I} \{{W}_{x;\psi }(\tau ,s)\}}{ {\mathcal R} \{{W}_{x;\psi }(\tau ,s)\}})$$

In many application, it is on interest to study and quantify the dependencies between two non-stationary time series. The cross-wavelet analysis provides appealing information such as the similarity between the wavelet power spectrum of the two series (computing the cross-wavelet power or the coherency) and the series’ synchronicity (estimating the phase differences at certain periods). In particular, the cross-wavelet power is

5 $${(XWP)}_{x,y}(\tau ,s)=|{W}_{x;\psi }(\tau ,s){W}_{y;\psi }^{\ast }(\tau ,s)|$$

where *W*_*x*;*ψ*_(*τ*, *s*) and *W*_*y*;*ψ*_(*τ*, *s*) are CWT (2) of the functions *x*(*t*), $y(t)\in {\mathbb{R}}$ and their product is the cross-wavelet transformation. The wavelet power spectrum (3) can be interpret as the local variance of the time series; instead, the cross-wavelet power spectrum (5) represents the local covariance between the two compared time series for each time and frequency. So, the cross-wavelet power quantify the similarity of power between two series.

The global cross-wavelet power at the scale s, is defined as the time-average of (5), and it measures the cross-covariance between two time series as a function of frequency. Furthermore, the phase-difference of *x*(*t*) over *y*(*t*) is

6 $${\varphi }_{x,y}(\tau ,s)=(\frac{ {\mathcal I} \{{W}_{x,y}(\tau ,s)\}}{ {\mathcal R} \{{W}_{x,y}(\tau ,s)\}})$$

and (6) is equal to the difference between *ϕ*_*x*_(*τ*, *s*) − *ϕ*_*y*_(*τ*, *s*). A phase-difference of zero indicates that the time series move together at a specific time-frequency, an absolute value less (greater) than *π*/2 indicates that the two series move in phase (anti-phase) and the positive (negative) sign of (6) shows that *y*(*t*) leads *x*(*t*) (*x*(*t*) leads *y*(*t*)). Usually a average phase difference over the time period for a selected period band is computed.

Statistical inference is performed to assess the null hypothesis of no periodicity (for wavelet (3)) and this test is based on Monte Carlo simulations, for further details see^45–47^.

In this paper, the wavelet transformations, as well as the statistical inference, are computed with the WaveletComp package^48^ in the *R* statistical software^49^. The longer the series, the more robust are the results of the CWT, and it is appropriate to use sequence with high number of complete cycles of the longer investigated period. Nevertheless, if the sequence is not so long and truncated, this methodology still allows to detect the presence of persistence periodicity. For example, Figure S7a shows a simulated time series with 1000 observations and a periodicity every 30 and 80 observations. In Figure S7b, there is the global WPS considering the whole series, 500, and 100 observations. Although the magnitude is not the same in particular for the longer cycle, the peaks are clearly recognisable even when only 100 observations are considered. In our analysis, the length of the radon time series is different for all the sequences and also they are truncated with respect to the year cycle. In the results, we will detect local maxima and we also discuss the values of the WPS curves.

## Supplementary information

Supplementary material for 'Multiple seasonality in soil radon time series'

## Data Availability

Raw data is available upon request.
