# Supplementary material for: Multiple seasonality in soil radon time series
Source: Sci Rep. 2019 Jun 13;9:8610. doi: 10.1038/s41598-019-44875-z (PMC6565803; doi:10.1038/s41598-019-44875-z)
Supplement: Supplementary file 1 — Supplementary material for 'Multiple seasonality in soil radon time series' [file 41598_2019_44875_MOESM1_ESM.pdf]

Supplementary material for 'Multiple seasonality in soil radon time series'

Marianna Siino, Salvatore Scudero, Valentina Cannelli, Antonio Piersanti, Antonino D'Alessandro

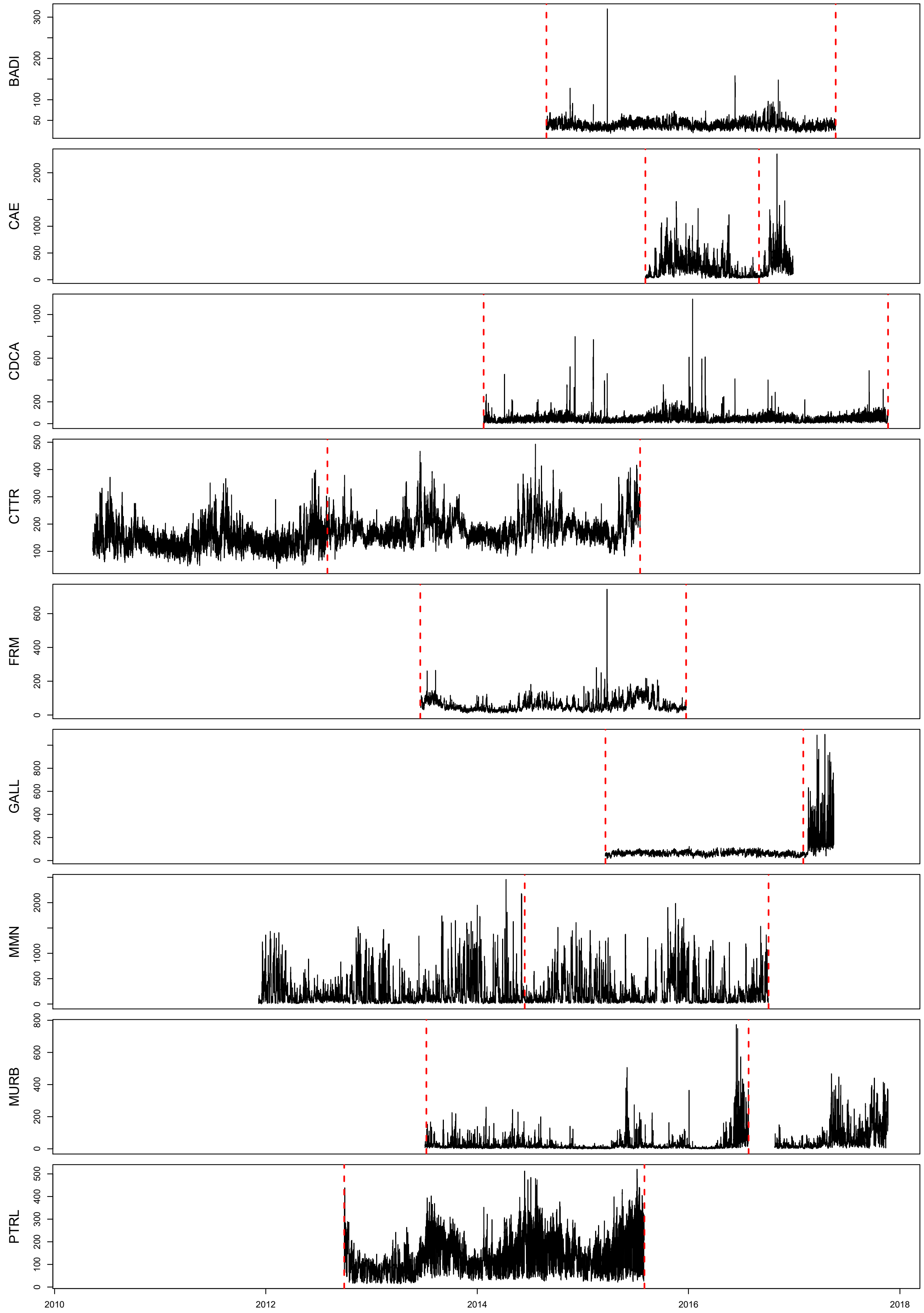

Figure 1: 2-hourly radon concentration time series for the 9 monitoring sites (values are in Bq/m^3). In the analysis, gaps shorter than 7 days have been filled with weighted moving average for both radon and temperature time series, instead for longer gaps the series has been properly cut (i.e. for CAE, MMN and MURB). CTTR and GALL the instruments have been relocated and then the time series have been accordingly cut. The vertical red lines delimit the period considered in the analysis.

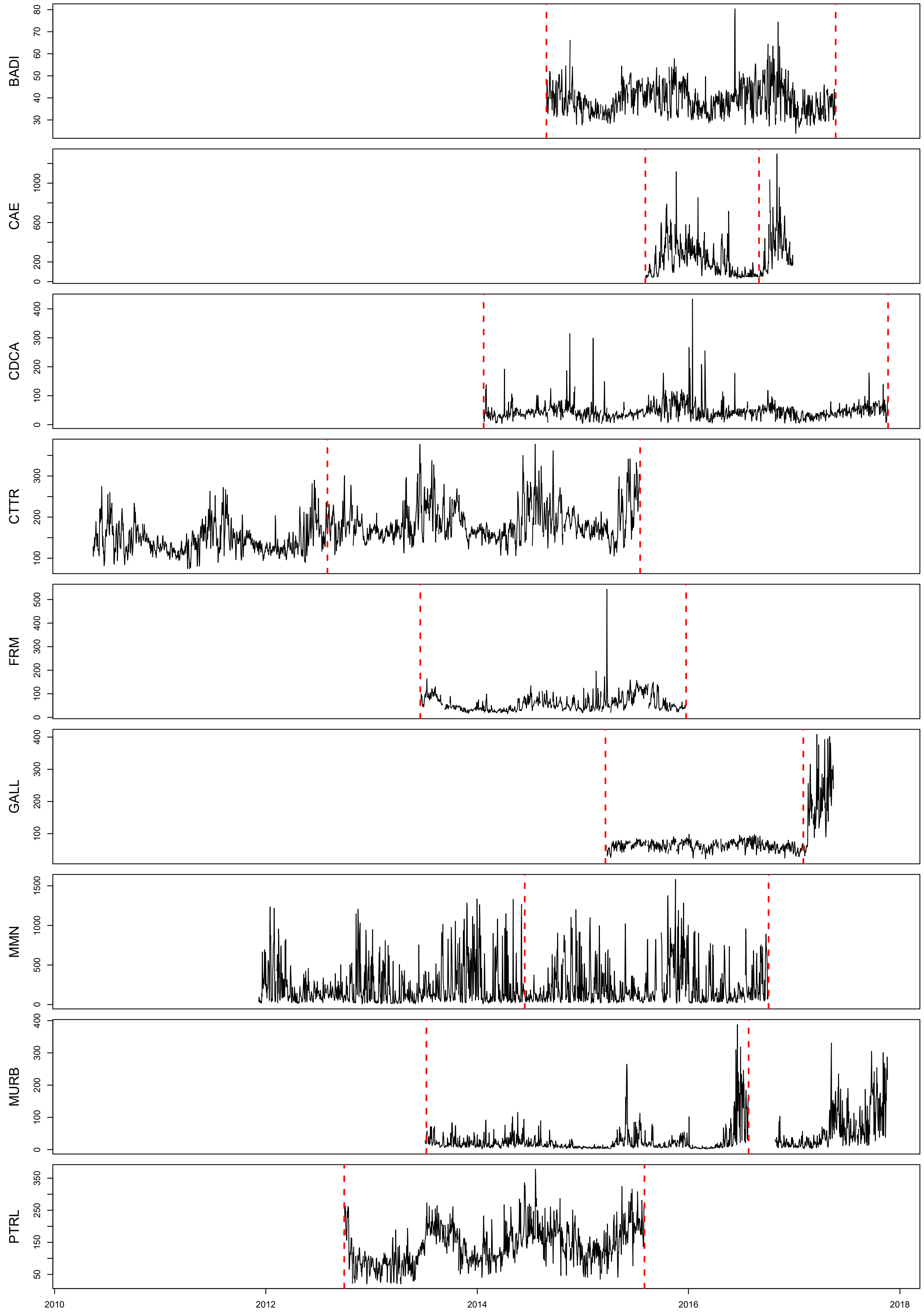

Figure 2: Mean daily radon concentration time series for the 9 monitoring sites (values are in Bq/m^3). In the analysis, gaps shorter than 7 days have been filled with weighted moving average for both radon and temperature time series, instead for longer gaps the series has been properly cut (i.e. for CAE, MMN and MURB). CTTR and GALL the instruments have been relocated and then the time series have been accordingly cut. The vertical red lines delimit the period considered in the analysis.

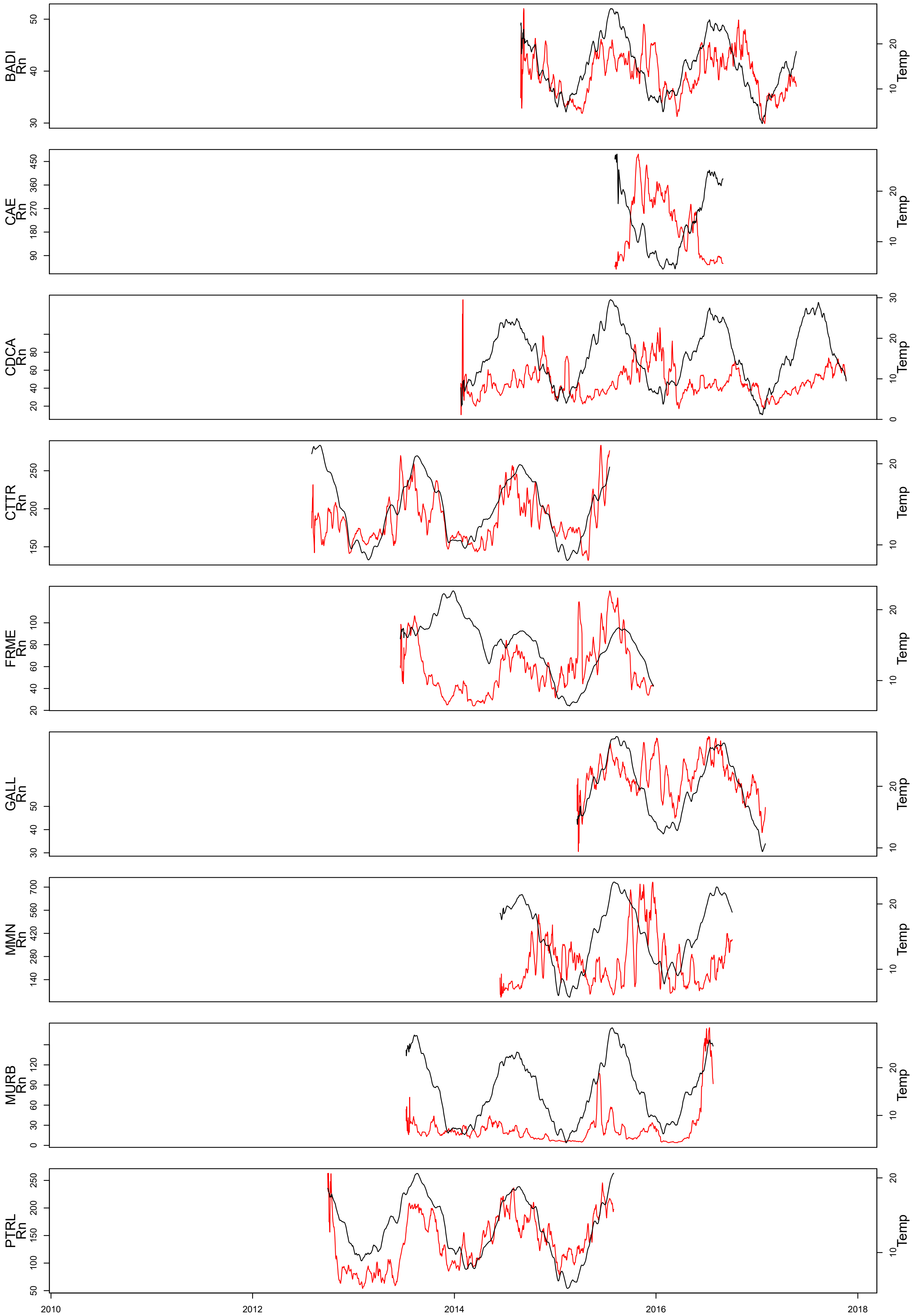

Figure 3: For each site, the 15-days moving average radon concentration time series (Bq/m^3, red lines) and the 15-days moving average temperature (C^o, black lines).

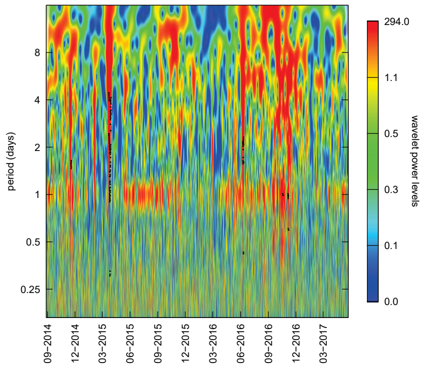

(a) BADI

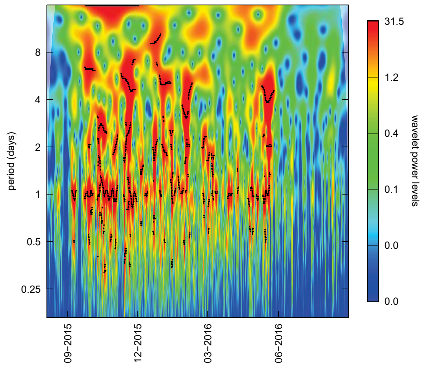

(b) CAE

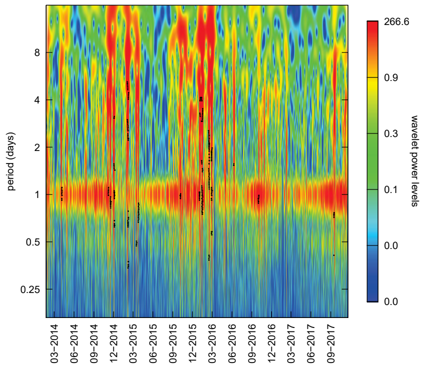

(c) CDCA

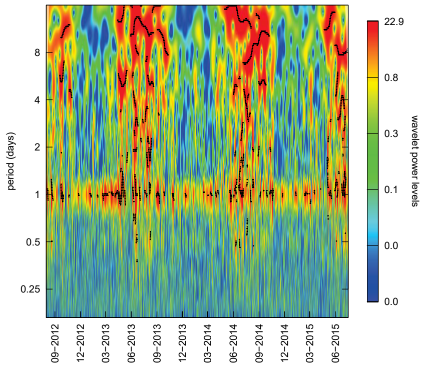

(d) CTTR

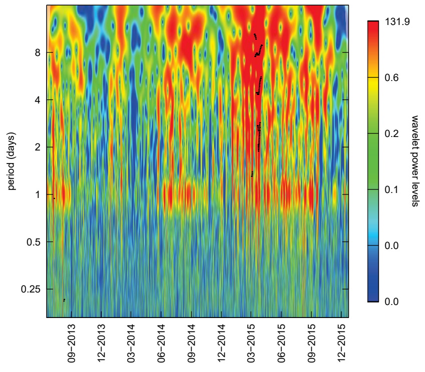

(e) FRME

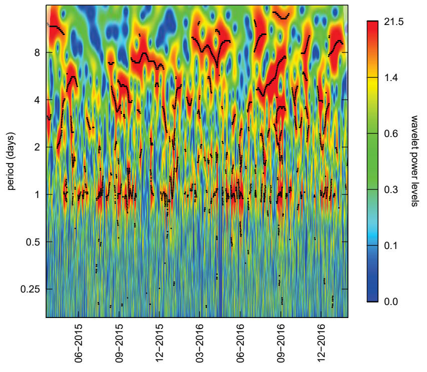

(f) GALL

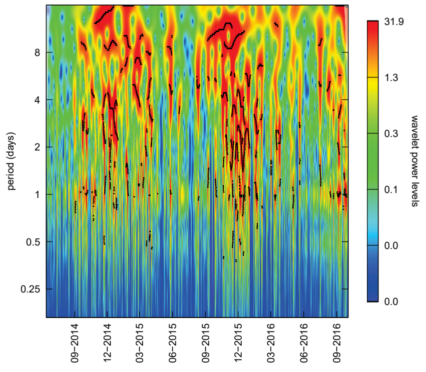

(g) MMN

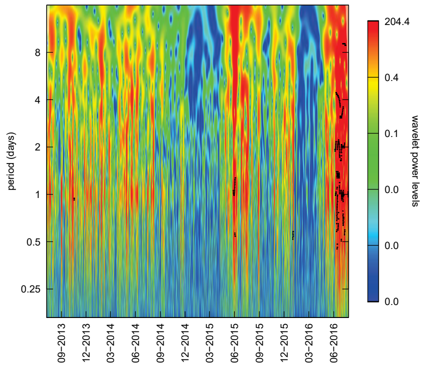

(h) MURB

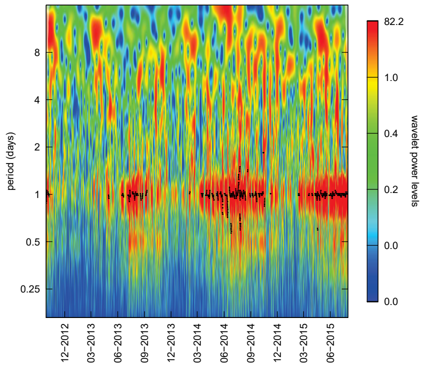

(i) PTRL

Figure 4: Wavelet power spectrum of 2-hours radon series in time-frequency domain with the CWT method. The black contour indicates the significant period with 90% confidence level. The lighter shade is the regions influenced by edge effects. The corresponding power spectrum density marginalising over time is in Figure5.

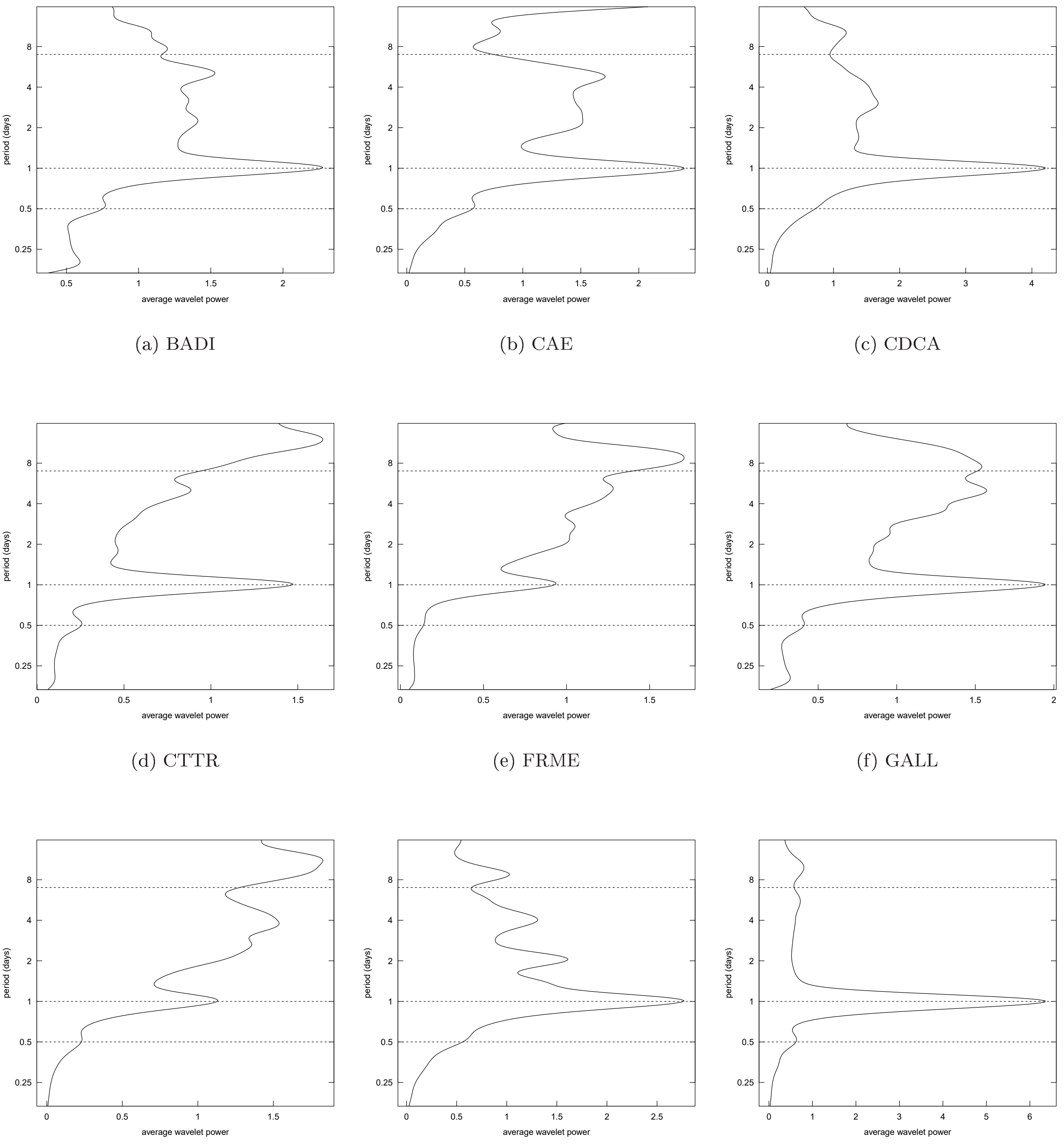

(a) BADI (b) CAE (c) CDCA

(d) CTTR (e) FRME (f) GALL

(g) MMN (h) MURB (i) PTRL

Figure 5: Global power spectrum density for the two-hourly radon measurements. The horizontal lines are for the 0.5-day, 1-day and 7-day periods.

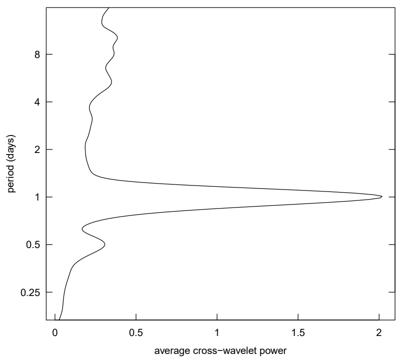

(a) BADI

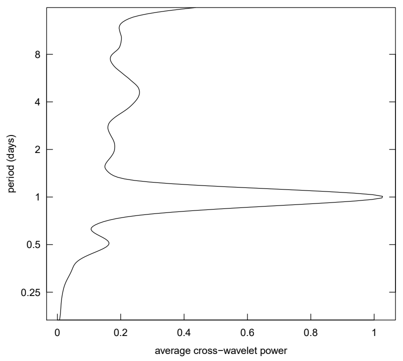

(b) CAE

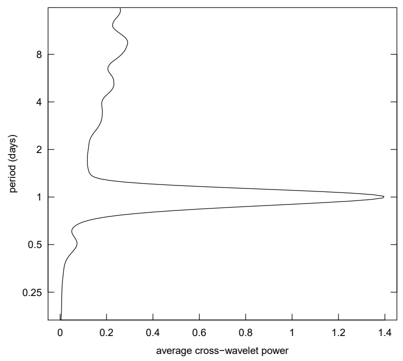

(c) CDCA

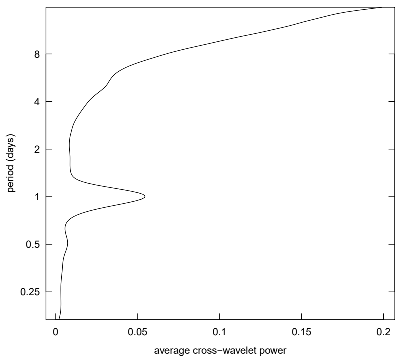

(d) CTTR

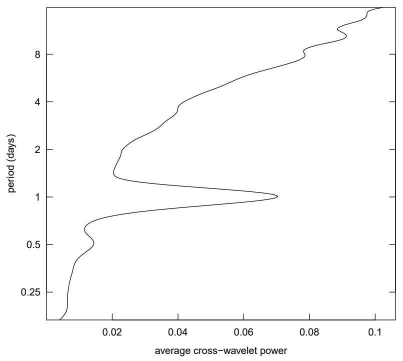

(e) FRME

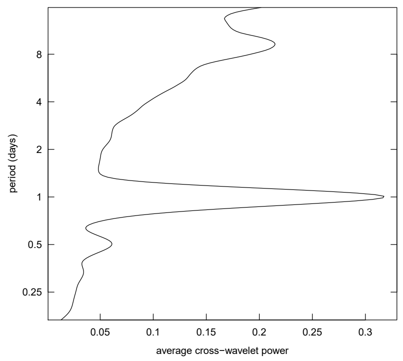

(f) GALL

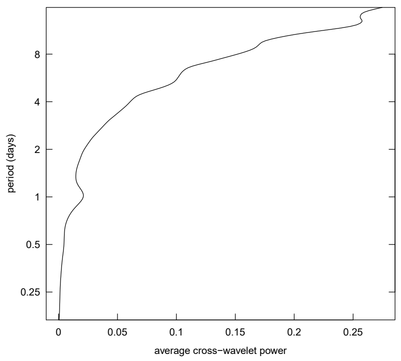

(g) MMN

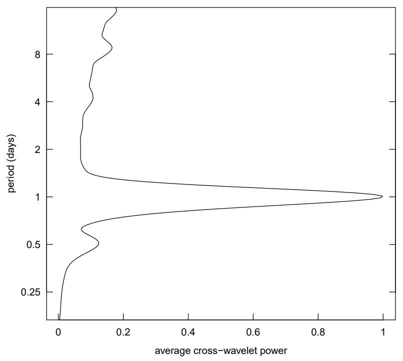

(h) MURB

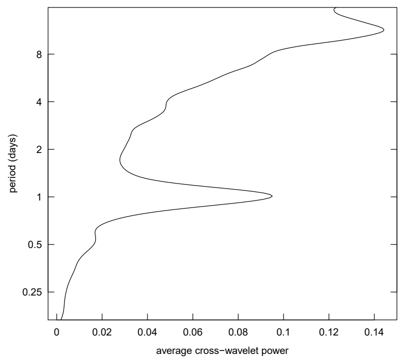

(i) PTRL

Figure 6: Global cross-power spectrum of 2-hourly radon and temperature time series.

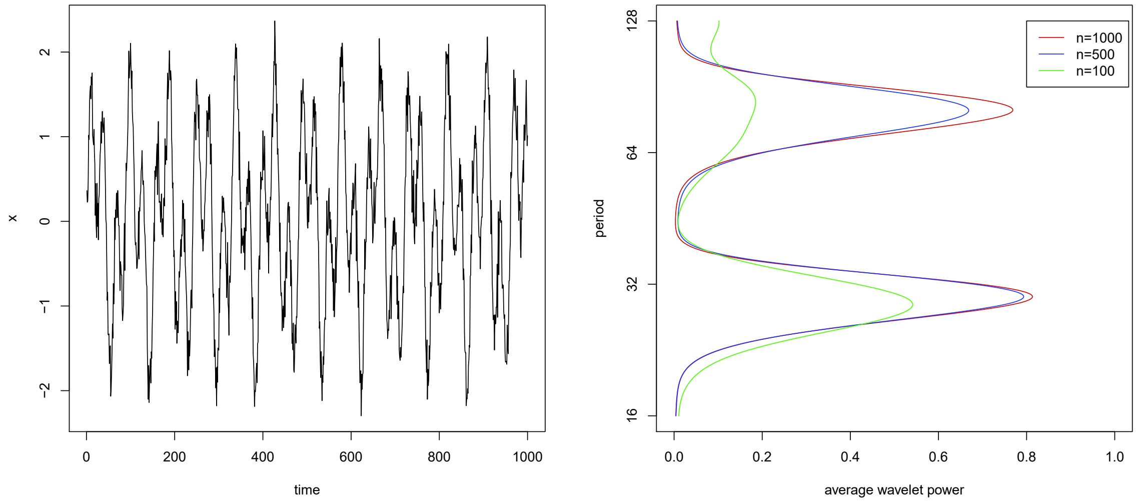

(a) (b)

Figure 7: 7a simulated time series of 1000 observations with main periodicities at 80 and 30 samples. 7a, global wavelet power spectrum density of the entire series (red line) and for two subsets of 500 and 100 observations (blue and green lines respectively).
